# Cefepime-Induced Neurotoxicity in an Elderly Patient: A Case Report

**DOI:** 10.7759/cureus.95713

**Published:** 2025-10-29

**Authors:** Miguel Herrera, Carlos F Caballero, Brian J Robles, Karen Courville

**Affiliations:** 1 Internal Medicine, Hospital Gustavo Nelson Collado, Chitre, PAN; 2 Research and Development, Sistema Nacional de Investigación, Panama, PAN; 3 Research, Instituto de Ciencias Médicas, Las Tablas, PAN; 4 Nephrology, Hospital Gustavo Nelson Collado, Chitre, PAN

**Keywords:** cefepime-induced neurotoxicity, chronic kidney disease (ckd), drug-related side effects and adverse reactions, elderly people, neurological manifestations, symptom assessment

## Abstract

Cefepime, a fourth-generation cephalosporin, may cause neurotoxicity, especially in patients with renal impairment. We report an 81-year-old man with chronic kidney disease (CKD) treated with intravenous cefepime for pneumonia. After five days, he developed acute confusion and reduced consciousness. Laboratory results showed no metabolic abnormalities and no microbial growth in blood cultures, and chest X-ray images improved.

Cefepime-induced neurotoxicity (CIN) was suspected; the drug was discontinued and replaced with piperacillin-tazobactam. Neurological status recovered fully within 48 hours. This case underscores the need to recognize cefepime neurotoxicity in elderly patients with renal dysfunction, ensuring timely intervention through dose adjustment or discontinuation.

## Introduction

Cefepime is a fourth-generation cephalosporin widely used for severe bacterial infections due to its broad-spectrum coverage and stability against β-lactamases. Although generally well tolerated, cefepime has been increasingly associated with neurotoxicity, particularly among older adults and patients with renal impairment. The reported incidence of cefepime-induced neurotoxicity (CIN) varies between 1% and 15%, depending on population risk and dosing practices [1,2]. CIN typically presents with altered mental status, confusion, myoclonus, or seizures, and may mimic other causes of encephalopathy, such as sepsis-associated or uremic encephalopathy [3].

The pathophysiology of CIN is not completely understood but is believed to involve inhibition of γ-aminobutyric acid (GABA) receptors and accumulation of cefepime due to impaired renal clearance [4,5] and has been linked to dose-dependent inhibition of GABAergic neurotransmission, leading to neuronal hyperexcitability, especially when serum concentrations exceed 20 mg/L. Recent literature underscores the importance of early recognition, appropriate dose adjustment in renal dysfunction, and discontinuation of cefepime to ensure full recovery [6].

Pharmacovigilance analyses estimate that cefepime-induced neurotoxicity may account for approximately 1%-3% of all antibiotic-related neurological adverse events and up to 15% among critically ill patients with renal dysfunction [7]. Mortality associated with delayed recognition has been reported in up to 10%-15% of severe cases, underscoring its clinical importance [8]. The most commonly reported manifestations include altered consciousness, myoclonus, aphasia, and seizures, which may appear within 2-5 days of drug initiation and resolve after discontinuation or hemodialysis [9].

The clinical recognition of this condition remains challenging, as symptoms overlap with other etiologies and laboratory results may not provide clear differentiation. The main differential diagnoses to consider include sepsis-associated encephalopathy, metabolic encephalopathy, uremic encephalopathy, and stroke-related causes, which share overlapping clinical features but differ in onset and reversibility [10].

## Case presentation

An 81-year-old male with a history of hypertension, type 2 diabetes mellitus, cerebrovascular disease with motor sequelae, and stage 3b chronic kidney disease (CKD) presented to the emergency department of a second-level hospital in Panama (June 2025) with fever, dry cough, dyspnea, and confusion. Two weeks earlier, he had been hospitalized for 10 days at the same facility for influenza B pneumonia, treated with oseltamivir.

On admission, his blood pressure was 140/72 mmHg, heart rate was 54 bpm, respiratory rate was 22 breaths per minute, temperature was 38.2°C, and oxygen saturation was 90% on room air. Physical examination revealed subcostal retractions and bibasilar crackles. Chest radiography showed bilateral pericardial consolidations. He was admitted due to severity criteria with oxygen requirement. Given the recent viral infection that had required hospitalization and the data of elevated white blood cells on admission, the infectious disease evaluation team recommended starting cefepime (1 g intravenously every 24 hours) for seven days due to suspected nosocomial pneumonia. Admission laboratory results are shown in Table 1.

**Table 1:** Laboratory findings on admission and on day 4, showing renal and metabolic abnormalities

Parameter	Admission	4th day	Reference range
Hemoglobin	12.2 g/dL	11.8 g/dL	13-17 g/dL
White blood cells	12.86 × 10⁹/L	10.45 × 10⁹/L	4-10 × 10⁹/L
Platelets	216 × 10⁹/L	224 × 10⁹/L	150-400 × 10⁹/L
Sodium	130 mmol/L	136 mmol/L	135-145 mmol/L
Potassium	5.15 mmol/L	4.9 mmol/L	3.5-5.1 mmol/L
Urea nitrogen	26 mg/dL	24 mg/dL	<20 mg/dL
Creatinine	1.59 mg/dL	1.63 mg/dL	0.6-1.3 mg/dL
C-reactive protein	50.5 mg/L	22 mg/L	<0.55 mg/L
Procalcitonin	0.31 ug/L	0.1 ug/L	<0.1 ug/L
Glucose	140 mg/dL	129 mg/dL	70-110 mg/dL

**Figure 1:**
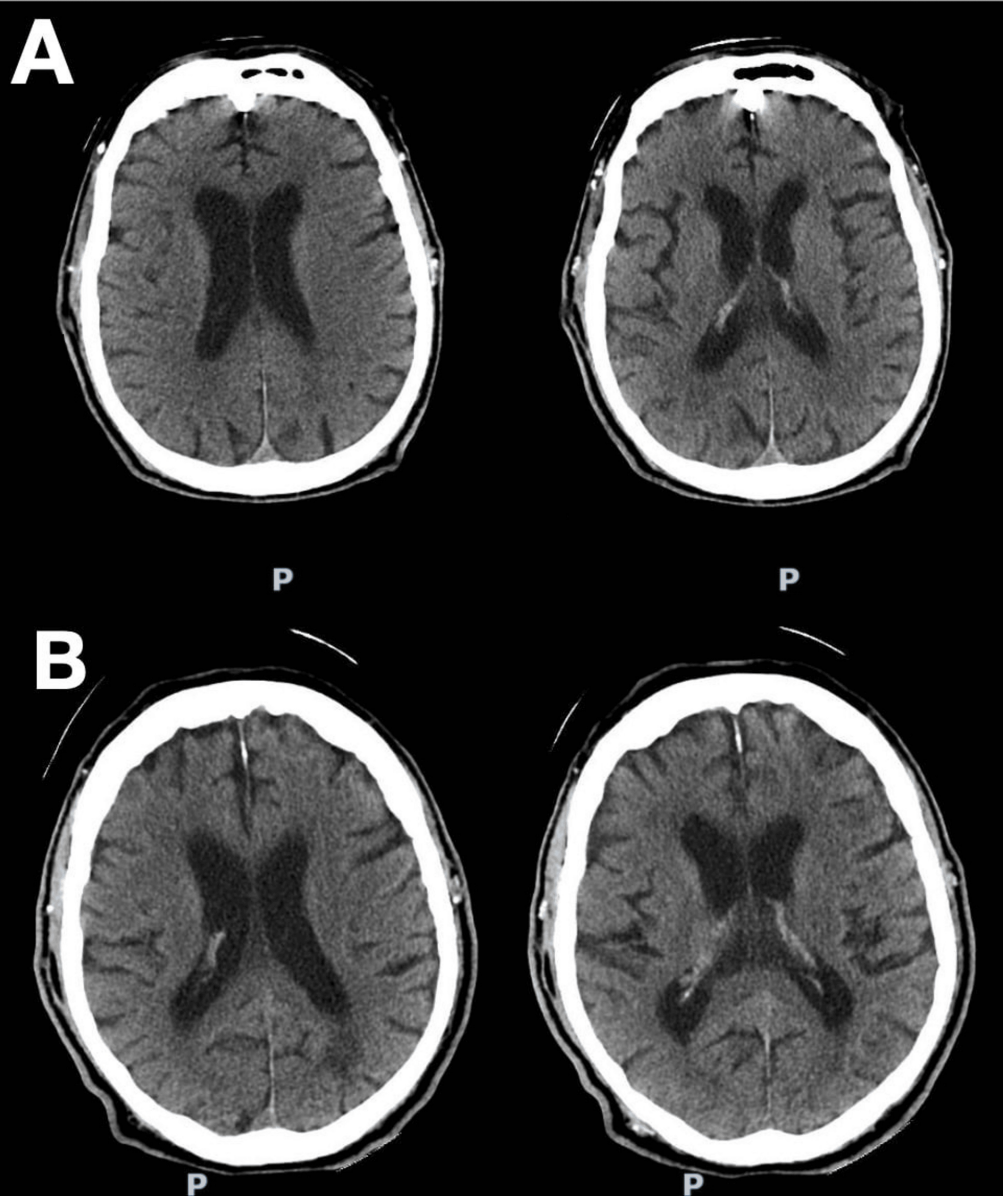
Non-contrast brain CT scans (A) Initial scan with no evidence of hemorrhage or acute ischemia. (B) Follow-up at 48 hours showing no ischemic lesions. CT: computed tomography

During the first few days, his clinical course was favorable, with the disappearance of fever and decreased oxygen requirement. On the fourth day of treatment, he developed lethargy and disorientation. Neurological examination revealed fluctuating lethargy with intermittent confusion, prompting a non-contrast brain computed tomography (CT) scan, which showed no ischemic lesions, hemorrhage, tumors, or other abnormalities (Figure 1).

**Figure 2:**
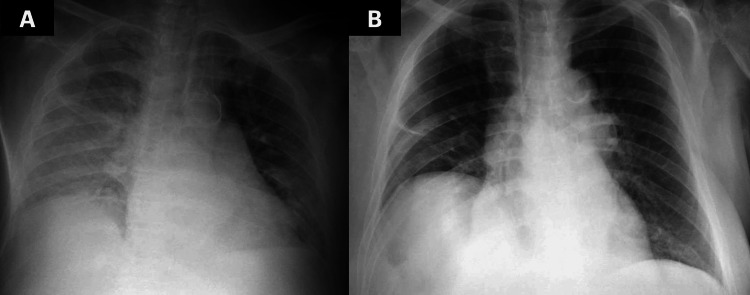
Chest radiographs from admission (A) and fifth day of hospitalization (B)

Blood and urine cultures were negative, and the patient was afebrile. Laboratory tests on the fourth day showed decreased leucocyte count to the normal range and no electrolyte abnormalities. Kidney function remained at 43 mL/min/m^2^. Chest radiographs revealed improvement of the consolidations (Figure 2). Cefepime was discontinued on the fifth day due to suspected neurotoxicity, and piperacillin/tazobactam (4.5 g every six hours) was initiated to complete the initially recommended treatment course.

Magnetic resonance imaging (MRI) and electroencephalography (EEG) were unavailable; therefore, a follow-up CT scan was performed 48 hours later, showing no changes (Figure 1). The patient completed seven-day antibiotic therapy and experienced progressive neurological improvement, with resolution of confusion and full recovery of alertness on day 8. He was discharged without disorientation on day 9, with clinical and laboratory improvement and no oxygen supplementation.

## Discussion

Cefepime-induced neurotoxicity is an increasingly recognized adverse effect, particularly in patients with renal impairment, where drug clearance is reduced. A systematic review by Payne et al. reported that approximately 85% of cases occurred in individuals with some degree of renal dysfunction [2]. The onset of neurotoxicity symptoms typically occurs within 2-5 days of initiating therapy and resolves within 2-3 days after discontinuation or dose adjustment [4,8]. The temporal relationship between drug exposure and symptom evolution strongly supports cefepime as the causative agent: neurocognitive changes emerged on day 4 of therapy, improved within 48 hours of discontinuation, and resolved completely by discharge.

The mechanisms of cefepime neurotoxicity are primarily related to excessive plasma and cerebrospinal fluid concentrations, leading to GABA-A receptor antagonism and neuronal hyperexcitability [7]. In patients with CKD, impaired excretion prolongs cefepime’s half-life, allowing for toxic accumulation even with conventional dosing. Advanced age further contributes to reduced renal function and increased pharmacodynamic sensitivity [11].

Clinical manifestations can include confusion, agitation, aphasia, myoclonus, seizures, and coma [12]. After ruling out structural, metabolic, and infectious causes, cefepime-induced neurotoxicity was considered. This diagnosis is often overlooked and should be contemplated, particularly in patients with CKD (especially with glomerular filtration rates < 30 mL/min/1.73 m²), age ≥ 70 years, neurological diseases, hypertension, and diabetes mellitus [13] with unexplained neurological changes.

To strengthen this clinical association, the Naranjo Adverse Drug Reaction Probability Scale was applied, yielding a score of 7, indicating a probable adverse drug reaction [14]. Likewise, under the WHO-UMC causality assessment, the relationship was classified as probable/likely based on the temporal sequence and absence of alternative explanations [15].

Recent studies have emphasized the need for dose adjustment based on glomerular filtration rate and regular therapeutic monitoring in high-risk patients [16]. Prevalence of chronic kidney disease is 10% of the global population [17]. Awareness among clinicians is crucial since timely discontinuation of cefepime typically results in rapid recovery, avoiding unnecessary neuroimaging or invasive diagnostic tests. These findings align with previously reported timelines in which neurotoxicity typically develops within the first week of cefepime administration and resolves after cessation or dialysis [18]. Appropriate dosing vigilance, renal function monitoring, and interprofessional awareness are key to preventing cefepime-related neurotoxicity.

## Conclusions

Cefepime-induced neurotoxicity is a potentially reversible but underrecognized complication of antibiotic therapy, especially in elderly patients with renal impairment. Early recognition of neurological symptoms, prompt cessation of cefepime, and appropriate dose adjustments based on renal function are essential for favorable outcomes. Clinicians should maintain a high level of suspicion when treating high-risk populations and ensure regular monitoring of kidney function during therapy. This case reinforces the importance of individualized antimicrobial dosing and multidisciplinary management to minimize preventable adverse drug events.
